# Tumor Infiltrating Lymphocytes Affect the Outcome of Patients with Operable Triple-Negative Breast Cancer in Combination with Mutated Amino Acid Classes

**DOI:** 10.1371/journal.pone.0163138

**Published:** 2016-09-29

**Authors:** Vassiliki Kotoula, Sotiris Lakis, Ioannis S. Vlachos, Eleni Giannoulatou, Flora Zagouri, Zoi Alexopoulou, Helen Gogas, Dimitrios Pectasides, Gerasimos Aravantinos, Ioannis Efstratiou, George Pentheroudakis, Kyriaki Papadopoulou, Kyriakos Chatzopoulos, Pavlos Papakostas, Maria Sotiropoulou, Irene Nicolaou, Evangelia Razis, Amanda Psyrri, Paris Kosmidis, Christos Papadimitriou, George Fountzilas

**Affiliations:** 1 Department of Pathology, Aristotle University of Thessaloniki, School of Health Sciences, Faculty of Medicine, Thessaloniki, Greece; 2 Laboratory of Molecular Oncology, Hellenic Foundation for Cancer Research/Aristotle University of Thessaloniki, Thessaloniki, Greece; 3 Molecular Diagnostics Laboratory, INRASTES, NCSR 'Demokritos', Athens, Greece; 4 DIANA-Lab, Department of Computer and Communication Engineering, University of Thessaly, Volos, Greece; 5 Victor Chang Cardiac Research Institute, Darlinghurst, New South Wales, Australia; 6 The University of New South Wales, New South Wales, Australia; 7 Department of Clinical Therapeutics, Alexandra Hospital, National and Kapodistrian University of Athens School of Medicine, Athens, Greece; 8 Department of Biostatistics, Health Data Specialists Ltd, Athens, Greece; 9 First Department of Medicine, Laiko General Hospital, National and Kapodistrian University of Athens School of Medicine, Athens, Greece; 10 Oncology Section, Second Department of Internal Medicine, Hippokration Hospital, Athens, Greece; 11 Second Department of Medical Oncology, Agii Anargiri Cancer Hospital, Athens, Greece; 12 Department of Pathology, Papageorgiou Hospital, Thessaloniki, Greece; 13 Department of Medical Oncology, Ioannina University Hospital, Ioannina, Greece; 14 Oncology Unit, Hippokration Hospital, Athens, Greece; 15 Department of Pathology, Alexandra Hospital, Athens, Greece; 16 Department of Histopathology, Agii Anagriri Cancer Hospital, Athens, Greece; 17 Third Department of Medical Oncology, Hygeia Hospital, Athens, Greece; 18 Division of Oncology, Second Department of Internal Medicine, Attikon University Hospital, Athens, Greece; 19 Second Department of Medical Oncology, Hygeia Hospital, Athens, Greece; 20 Aristotle University of Thessaloniki, Thessaloniki, Greece; University of Wisconsin Madison, UNITED STATES

## Abstract

**Background:**

Stromal tumor infiltrating lymphocytes (TILs) density is an outcome predictor in triple-negative breast cancer (TNBC). Herein we asked whether TILs are related to coding mutation load and to the chemical class of the resulting mutated amino acids, i.e., charged, polar, and hydrophobic mutations.

**Methods:**

We examined paraffin tumors from TNBC patients who had been treated with adjuvant chemotherapy mostly within clinical trials (training cohort, N = 133; validation, N = 190) for phenotype concordance; TILs density; mutation load and types.

**Results:**

Concordance of TNBC phenotypes was 42.1% upon local / central, and 72% upon central / central pathology assessment. TILs were not associated with mutation load, type and class of mutated amino acids. Polar and charged mutation patterns differed between TP53 and PIK3CA (p<0.001). Hydrophobic mutations predicted for early relapse in patients with high nodal burden and <50% TILs tumors (training: HR 3.03, 95%CI 1.11–8.29, p = 0.031; validation: HR 2.90, 95%CI 0.97–8.70, p = 0.057), especially if compared to patients with >50% TILs tumors (training p = 0.003; validation p = 0.015).

**Conclusions:**

TILs density is unrelated to mutation load in TNBC, which may be regarded as an unstable phenotype. If further validated, hydrophobic mutations along with TILs density may help identifying TNBC patients in higher risk for relapse.

## Introduction

Triple negative breast cancer (TNBC) is a diagnosis by exclusion corresponding to tumors that are immunohistochemically (IHC) negative for estrogen receptor (ER), progesterone receptor (PgR) and HER2 protein overexpression [1]. TNBC are in fact a variety of diseases under the same umbrella and as such, are characterized by extensive histological, phenotypic and genetic diversity [2]. The majority of TNBC are basal-like breast carcinomas (BLBC). The two terms are not interchangeable although they share a 70–80% overlap. BLBC are diagnosed as an intrinsic breast cancer subtype with widely used classifiers [3], while TNBC are called by ER/PgR/HER2 IHC as a proxy for BLBC [4]. In the clinic, in comparison to patients with other breast cancer subtypes for whom survival has improved or is expected to further improve with the application of disease-specific drugs, survival of patients with TNBC is still generally poor with yet no therapeutic options other than cytotoxic chemotherapy.

At the genomic level, TNBC are characterized by early structural aberrations that persist through clonal evolution and by a high incidence of TP53 mutations [5–9]; TNBC mutation load is more than ten times higher as compared to luminal carcinomas [9] but, with the exception of TP53, mutations and mutated genes are non-recurrent among tumors and may be heterogeneously affected within the same tumor [6, 9]. Research is currently ongoing for translating the huge amount of genomic data into clinical benefit for TNBC patients with no conclusive results yet.

In comparison to luminal subtypes, TNBC are also characterized by relatively higher rates of stromal tumor lymphocytic infiltrates (TILs) that reflect host anti-tumor immune response [10, 11] and confer better prognosis in the adjuvant [12–15] and neoadjuvant settings [16–18]. Irrespectively of their clonal frequency and impact on tumor maintenance, mutations producing neoantigens have been implicated in triggering host immune response against tumors [19]. TNBC are rich in mutations and in TILs, which may indicate interdependence of the two parameters, as has been shown for high mutational loads in other tumors arising in immunogenic environments, e.g., lung cancer, melanoma, cancers within the Lynch syndrome [19, 20]. Such associations have not yet been addressed in tumors arising in non-immunogenic environments, such as in the breast.

Predicting which mutations, provided that they are expressed within mutant epitopes, will result in triggering CD8 mediated cancer cell killing remains a still unmet challenge. The chemical nature of resulting mutant amino acids (charged, polar, hydrophobic) and epitopes might provide a hint in this direction. It was early recognized that preserved mutations during protein evolution preferentially occur for similar amino acid classes [21], because this way self-distraction would not be triggered. Further, polar-to-hydrophobic substitutions were identified as mutations evoking TILs response in patients with melanomas [22]. Supporting the above, hydrophobicity may provide a basis for T cells to recognize alien epitopes as immunogenic [23].

In a first attempt to approach the above issues at the tumor tissue level, herein we asked whether mutational load and the chemical nature of the resulting mutant amino acids in TNBC is associated with TILs density affecting the outcome of patients with operable disease. We hypothesized that mutant amino acid classes might interfere with patient outcome according to TILs density in the tumor. To this end, we examined mutation characteristics and TILs, as well as standard clinico-pathological parameters that affect early TNBC patient outcome, such as nodal status and tumor size, in two independent patient cohorts with informative targeted massively parallel sequencing data (MPS). Patients had mostly been treated within adjuvant clinical trials by the Hellenic Cooperative Oncology Group (HeCOG). Because the cohorts had been selected at different time points we also compared phenotype concordance upon local and central pathology testing; in addition, intra-tumoral (spatial) genetic heterogeneity was examined in different samples from different paraffin block depths from the same tumor.

## Materials and Methods

### Patients, tumors and samples

In the present retrospective/prospective translational study, two independent series of TN tumor tissues with informative targeted massively parallel sequencing (MPS) data were compared for the presence of variants and mutations, and were examined for patient outcome. Routinely processed TNBC tissues (FFPE) with annotated patient data were retrieved at two different time points from the HeCOG tissue bank and clinical databases; central histological review, phenotypic reassessment, and MPS were implemented in the Laboratory of Molecular Oncology (MOL; Hellenic Foundation for Cancer Research / HeCOG / Aristotle University of Thessaloniki, Thessaloniki, Greece). In total, 365 patients that had been diagnosed as TNBC locally and/or centrally were examined. Cohort (A), which served as the training set, included prospectively collected tumor blocks for biomarker analysis from TNBC patients treated in a series of adjuvant clinical trials by HeCOG, i.e., HE 10/00, HE 10/04A, HE 10/04B, HE 10/05, HE 10/08, and HE 10/10, as described in S1 Table or from patients who were ineligible to enter these trials and were routinely treated in HeCOG affiliated clinical centers. The 133 cases included in this cohort were selected based on tumor phenotype concordance in local and central pathology laboratories. Cohort (B) included tumors that had been characterized as TNBC upon local testing from patients treated only within the HE 10/97, HE 10/00, HE 10/05, and HE 10/08 trials (S1 Table). This cohort included 190 tumors and was used as a validation set. Patient characteristics for the two cohorts are shown in Table 1. For 82 cases in the validation cohort (S2 Table), targeted massively parallel sequencing (MPS) data from at least 2 DNA samples from the same tumor (76 from the same paraffin block; 6 from different blocks) were available [24]; these data were used for studying spatial heterogeneity within tumors. The study outline is summarized **in Figure A in** S1 File.

**Table 1:** Patient and tumor characteristics in the two study cohorts.

Patients	training	validation	p-value
N	133	190	
**Age (years)**			
Mean (SD)	50.7 (12.9)	52.2 (12.4)	0.21
Median	49	53	
Min-Max	28–77	21–83	
**Ki67**			
Mean (SD)	53.4 (31.1)	49.8 (32.6)	0.30
Median	52	55	
Min-Max	0–100	0–100	
	**N (%)**	**N (%)**	
**Age (years)**			
≤50	68 (51.2)	74 (39.0)	0.030
>50	65 (48.8)	116 (61.0)	
**Menopausal status**			
Postmenopausal	64 (48.2)	103 (54.2)	0.28
Premenopausal	69 (51.8)	87 (45.8)	
**Tumor size**			
≤2	43 (32.6)	72 (37.8)	0.33
>2	89 (67.4)	118 (62.2)	
**Positive lymph nodes**			
0–3	91 (70.0)	122 (64.2)	0.28
≥4	39 (30.0)	68 (35.8)	
**Histological grade**			
I	2 (1.6)	4 (2.2)	0.60
II	20 (15.0)	36 (19.0)	
III	111 (83.4)	150 (79.0)	
**Histological type**			
Medullary	6 (4.6)	16 (8.4)	0.29
NST	112 (84.2)	148 (77.8)	
Other	15 (11.2)	26 (13.6)	
**Surgery (binary)**			
MRM	57 (42.8)	99 (52.2)	0.10
Other	76 (57.2)	91 (47.8)	
**Hormonotherapy**			
No	107 (80.4)	160 (85.1)	0.27
Yes	26 (19.6)	28 (14.9)	
**Radiotherapy**			
No	34 (25.6)	44 (23.7)	0.70
Yes	99 (74.4)	142 (76.3)	
**ER/PgR/HER2 local**			
Either positive	0 (0.0)	0 (0)	
TNBC	133 (100)	190 (100)	
**ER/PgR/HER2 central**			
Either positive	0	80 (42.2)	
TNBC	133 (100)	97 (51.0)	
**Basal**			0.050
Yes	110 (84.0)	133 (74.7)	
No	21 (16.0)	45 (25.3)	
**Survival data**			
Median FU in months	79	70	
N of valid cases	133	190	
Deaths, N	35	39	0.070
Event free at 3 years, %	86,6	88,4	
Event free at 5 years, %	76,7	82,9	
Relapse, N	44	47	0.051
Event free at 3 years, %	75,7	82,6	
Event free at 5 years, %	71,2	77,2	

Notes: N: number; MRM: modified radical mastectomy; NST: non-specific type; FU: follow-up.

Patients had provided written consent for the use of their biologic material for research purposes and the study was approved by the Bioethics Committee of the Aristotle University of Thessaloniki School of Medicine (#77/10June2014) and by the Institutional Review Board of Papageorgiou Hospital of Thessaloniki (#725/10May2013). TNBC had been prospectively diagnosed in local pathology laboratories as ER/PgR/HER2 IHC negative.

Central tumor assessment at MOL included histology review; ER/PgR/HER2/Ki67 IHC and HER2 FISH where needed [25] by adapting HER2 evaluation according to the more recent ASCO/CAP guidelines [26]. Briefly, ER and PgR cut-off was 1% positive nuclei; HER2 was considered overexpressed for intense circumscribed staining in >10% tumor cells and HER2 gene amplification for HER2/CEP17 ratio ≥2 or for ≥6 HER2 copies; Ki67 was assessed as % positive nuclei (continuous variable); and, CK5 and EGFR were applied for typing basal-like carcinomas with 1% cut-off for positivity [27]. IHC and FISH were performed on in-house low-density tissue microarrays (TMA) that contained two 1.5mm cores per tumor. TMA H&E sections were also evaluated for tumor cell content (TCC%; tumor nuclei vs. all nuclei). TMAs for the two cohorts were constructed at independent time points but phenotyping was accomplished with the same cut-offs for both series; the evaluated tumor areas in overlapping cases were not identical for the same tumor. Such paired samples were used for the assessment of spatial heterogeneity. Stromal tumor infiltrating lymphocytes (TILs) density was also assessed on whole H&E sections based on Salgado et al [11], as previously described [13].

### MPS genotyping

DNA was extracted from TMA cores. The majority of samples (78%) had TCC ≥50% but samples with as low as 15% TCC were also processed. DNA was extracted with magnetic beads (VERSANT Tissue Prep Kit, Siemens Healthcare, Erlangen, Germany); quantity was measured with the Qubit fluorometer (Life Technologies, Paisley, UK); and, DNA amplification performance was evaluated by two different control qPCR assays. Criteria for processing samples for genotyping were ≥2ng/ul DNA amplifiable at Ct≤32 for two different qPCR control assays.

Genotyping was accomplished in an Ion Proton Sequencer; the two custom highly-multiplexed panels [24] targeted coding mutations and single nucleotide polymorphisms (SNPs) previously implicated in breast cancer [5, 7]. The T-panel was used in the training cohort and in 78 out of 82 cases with matched tumor samples; the B-panel was used in the validation cohort and in all matched samples (**Figure A in** S1 File). Samples were accepted for further evaluation if 90% of amplicons had been read >100 times and if >5 eligible variants were present. Variants obtained from Ion Reporter v.4 were considered ineligible for analysis if non-annotated, if indels with GC-stretches (reading artifacts with semiconductor sequencing); if position coverage <100 and if variant coverage <40. The mean and median reads for the analyzed variant positions were 894.3 and 509 with the T-panel in the training set; 1002.8 and 646 with the B-panel in the validation set.

For the purposes of the present study, coding mutations and common SNPs (minor allele frequency (MAF) >0.1%) that were targeted with both panels were examined; mutations corresponded to amino acid changing variants in coding regions for which no MAF was reported or, if registered SNPs, with MAF <0.1%. Pathogenicity of mutations was not further addressed, since, despite the numerous bioinformatics tools that have been proposed for this purpose [28], predicting functional implications of tumor mutations is still questionable [29].

Common gene targets with both panels were analyzed in the training and validation sets, and for the assessment of heterogeneity. For avoiding false heterogeneous calls, only positions that had been covered >100 times with both panels were compared from raw Ion Reporter data, as described in [24].

### Statistics

TIls were examined as a continuous variable and, for presentation purposes, as a 3-scale variable, 0–5%, 5–50% and >50%, whereby the latter represented lymphocyte-predominant (LP) breast cancer (BC), previously associated with most favorable outcome in TNBC [13].

Classic mutation types (missense, frameshift indels, nonsense) were examined along with the nature of the resulting mutated amino acids. Amino acids were classified as charged (Arg, Lys, Asp, Glu); polar (Gln, Asn, His, Ser, Thr, Tyr, Cys, Met, Trp); and, hydrophobic (Ala, Ile, Leu, Phe, Val, Pro, Gly). These were examined as single markers or in combined patterns that were present in tumors. Venn diagrams were plotted for overlapping features with Oliveros, J.C. (2007–2015) Venny. An interactive tool for comparing lists with Venn's diagrams. http://bioinfogp.cnb.csic.es/tools/venny/index.html

Classic descriptive statistics were applied (chi-square or Fisher’s exact test for categorical variables; Mann-Whitney or Kruskal-Wallis test for testing categorical against continuous variables). According to patient data origin, follow up periods ranged from 15 years for patients in the HE 10/00 trial down to 5 years in the more recent HE 10/10 trial (S1 Table). For this reason, only disease-free survival (DFS) was assessed in the present study. DFS was measured from the date of diagnosis until verified disease relapse or death, whichever occurred first, or loss from follow-up. Event free patients were censored at the date of last contact. Time-to-event distributions were compared by using Kaplan-Meier curves and log-rank tests. Univariate Cox regression analysis was used for reporting hazard ratios, while the Firth’s correction for monotone likelihood was used in the case of zero or few events. Survival status was updated in June 2014.

Regarding multivariate Cox regression models, the clinicopathological parameters were chosen by backward elimination among the following adjustment factors: age (>50 vs. ≤50 years), menopausal status (post vs. pre), tumor size (>2 cm vs. ≤2 cm), histological grade (I-II vs. III), adjuvant radiotherapy (yes vs. no), and Ki67 as a continuous variable by 5% increments. Positive nodes (≥4 vs. 0–3) and TILs density (LPBC vs. non-LPBC) were also added to models in the case they were not included in any combined variable.

All univariate tests were two-sided, with the significance level at α = 0.05, while significance threshold multivariate models was set at α = 0.15, a level higher than usual in order to control for bias in the estimations. No correction for multiple testing was made due to the exploratory nature of the study.

The analysis was fully compliant with the reporting recommendations for tumor marker prognostic studies [30]. The SAS software was used for statistical analysis (SAS for Windows, version 9.3, SAS Institute Inc., Cary, NC).

## Results

### Cohort specifications

The phenotypes of the 190 tumors in the validation cohort were TNBC upon local and central testing in 48.9%, locally TNBC / centrally nonTNBC in 42.1%, and locally TNBC without central assessment in 8.9% of the cases (**Figure A in** S1 File). Discordance concerned ER/PgR status, in accordance with previous reports even between central laboratories [31]. The incidence of basal carcinomas was significantly lower in locally TNBC / centrally nonTNBC (61.2%) as compared to concordant (86%) tumors (p<0.0001). The 82 tumors with ≥2 matched DNA samples that were also examined in the validation cohort had been phenotyped once locally as TNBC, and twice centrally at independent time points; 23 out of these tumors (28%) had one central call as nonTNBC upon heterochronous testing. Patient characteristics in this 82-case subset (S2 Table) did not differ from those in the actual study cohorts (Table 1).

### Genotyping results and coding mutation features

Among 2158 variants in all cases tested with the T-panel (133 in the training cohort and 78 in the matched series), 215 were coding mutations (10%) and 1657 were common SNPs (76.1%). Correspondingly, among 2304 variants in the 190 cases tested with the B-panel, 277 were mutations (12%) and 1697 common SNPs (74%). The mean and median numbers of mutations per tumor were similar with both panels (**Figure B in** S1 File). Coding mutation patterns in the two cohorts, i.e., mutation incidence and gene involvement were as expected for TNBC [5–7, 32]. However, the incidence of TP53 and PIK3CA mutations was significantly different between the training cohort and the locally/centrally discordant subset of the validation cohort (S3 Table). In the 82-case subset with ≥2 matched samples, data from 170 tissue and 12 blood samples were available for analysis (**Figure A in** S1 File). The results of matched genotype comparisons with the two panels are shown in **Figure C in** S1 File and in S4 Table. It appeared that (a) SNPs were more frequently conserved in matched samples than coding mutations; (b) coding mutations were common in half of the matched cases only (45% common TP53 mutations); (c) no two tumors had identical genotypes when taking into account both SNPs and coding mutations. Private mutations with either panel for the same tumor were examined in the validation cohort. Thus, the final number of mutations in the training and validation cohort was 149 and 257, respectively; these were distributed in 94/133 (70.7%) and in 125/190 (65.8%) cases.

**Fig 1:**
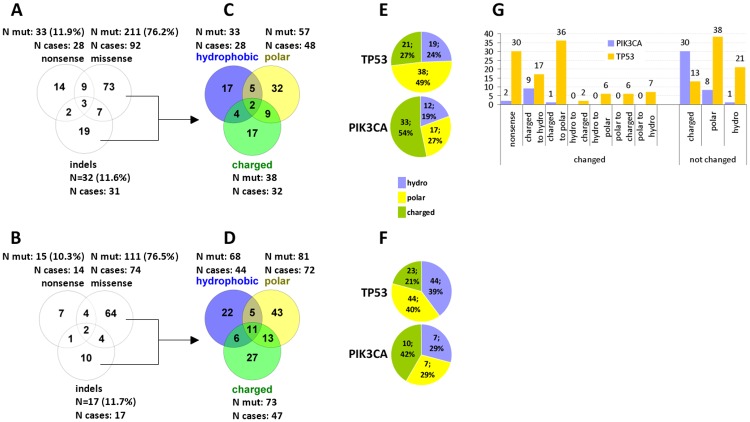
Coding mutation types and amino acid classes in TNBC. A, C, E: training cohort. B, D, F: validation cohort. The distribution of classic mutation types (A and B) and of mutated amino acid classes (C and D) did not differ between the two cohorts. Mutated amino acid classes in TP53 and PIK3CA showed the same distribution in the two cohorts but differed significantly for the two genes (E and F). The difference concerned both changed and not changed amino acid classes as compared to the reference (G, combined data for the two cohorts).

The type of mutations (missense, indels, nonsense) was strikingly similar, and the distribution of resulting charged, hydrophobic and polar amino acids was not statistically significantly different in the two cohorts (S5 Table). Multiple mutations per tumor were mostly missense (Fig 1A and 1B), which was particularly prominent in the hypermutated cases (22 and 60 mutations per case) in the validation cohort. Relatively few tumors had multiple types of mutations (11/94 [11.7%] and 18/127 [14.2%] in the training and validation cohort, respectively). In comparison, the rate of combinations of resulting mutated amino acid types was more than double in both cohorts (Fig 1C and 1D). The distribution of mutated amino acid types differed in TP53 and PIK3CA (Fig 1E and 1F); mutations in TP53 were more frequently polar and the mutated amino acid class was more frequently changed in comparison to PIK3CA mutations that resulted in charged amino acids without class change (Fisher’s exact p<0.0001) (Fig 1G).

### Mutation load and types of mutations are not associated with TILs density

**Fig 2:**
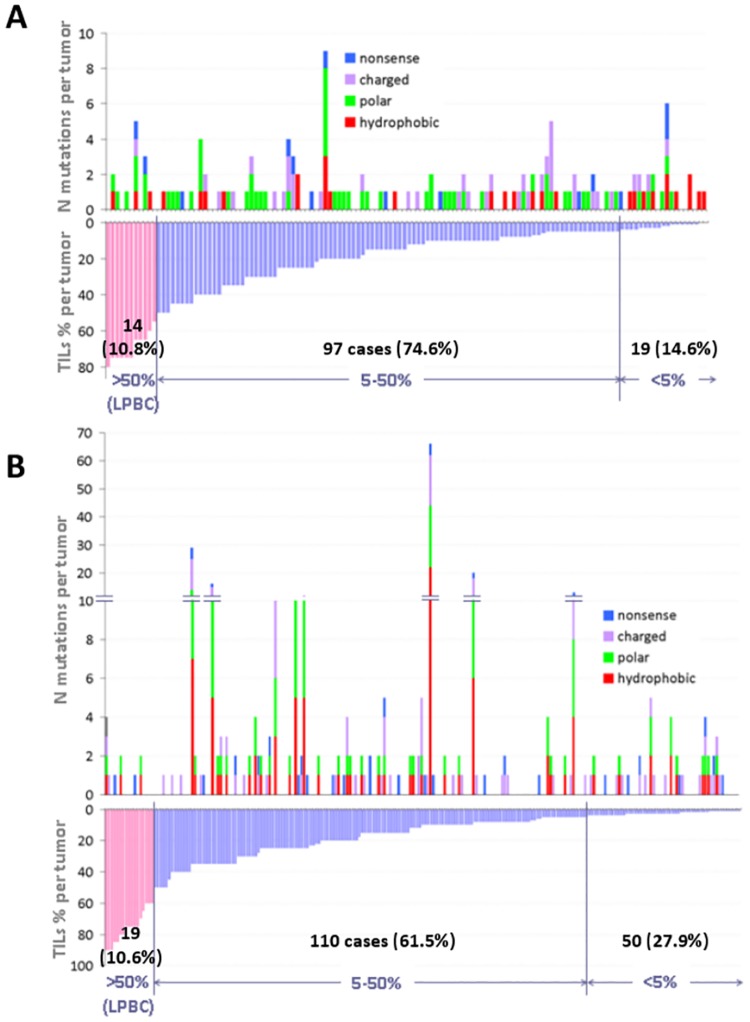
Coding mutations and mutated amino acid classes in association with TILs density. A: Training cohort, N cases = 130; B: Validation cohort, N cases = 179. The rate of LPBC was constant in the two cohorts, but the rate of tumors with <5% TILs was significantly higher in the validation cohort. The mutation load in LP tumors was similar to or lower than that observed in tumors with <5% TILs. No difference was observed in the distribution of mutation types according to TILs density.

It was possible to assess TILs in 130 and 179 tumors only, in the training and validation cohort, respectively, due to absence of adequate stroma in the available tumor sections. The rate of tumors with >50% TILs (LPBC) was constant, but the rate of tumors with <5% TILs, which was used as a lower cut-off for presentation purposes, was higher in the validation cohort (Fig 2A and 2B). Other than expected, LPBC had similar or lower mutation load and mutated genes than tumors with <5% TILs. In the training cohort, tumors with <5% TILs had significantly higher mutation load (mean 1.4 vs. 1.02) and numbers of mutated genes (mean 1.2 vs. 0.8) as compared to tumors with higher TILs (Mann-Whitney p = 0.0347 and p = 0.0302, respectively). No corresponding association was demonstrated in the validation cohort. Comparison of continuous TILs against the same mutation parameters did not yield significant associations either. In both cohorts, the rate of mutant tumors in LPBC was lower than the overall such rate. Because TP53 mutations were by far the most abundant in both TNBC cohorts, and also because unique mutation combinations with or without TP53 were observed, single gene comparisons against TILs would not yield meaningful results and were not applied. Classic types of mutations were not associated with TILs density, while nonsense mutations were also observed in LPBC.

The by definition lymphocyte-rich medullary carcinomas were particularly poor in mutations of any class; other than expected, hydrophobic mutations were more frequent in tumors with low TILs, which reached statistical significance in the training cohort (S6 Table). Single mutated amino acid classes were rare in LPBC, especially with regards to hydrophobic mutations. In the validation cohort, polar mutations were associated with larger tumor size (p<0.001), which was not significant in the training cohort. No further associations between mutated amino acid classes and clinicopathological parameters were observed.

### TILs density and hydrophobic mutations on patient DFS

Patient follow up period and the number of events in both cohorts is shown in Table 1. The effect of clinicopathological characteristics on DFS is shown in S7 Table. High nodal burden was the only parameter significantly associated with poor DFS in both cohorts. Mutations in single repeatedly affected individual genes were not associated with patient outcome (S8 Table). Risk probability analysis with respect to TILs density revealed an overall higher risk for relapse in the training as compared to the validation cohort but the effect of TILs on DFS was in the same direction (**Figure D in** S1 File).

**Fig 3:**
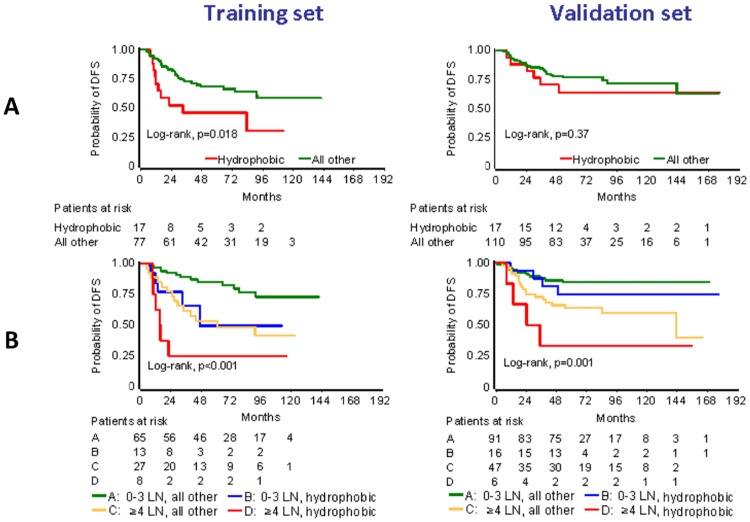
Hydrophobic mutations adversely affect patient DFS. Analysis was conducted in non-LPBC (tumors with <50% TILs). LN in B: nodal status. Patients with tumors with hydrophobic mutations performed worse in every context, in both cohorts.

Because of the rare incidence of single mutated amino acid classes in LPBC (Fig 2, S6 Table), the relevant analysis was applied for non-LPBC. The presence of charged and polar mutations, either as single parameters or as combined mutated amino acid classes, had no effect on patient outcome in both cohorts. In the training cohort, the presence of only hydrophobic mutations was strongly associated with poor DFS, whereby 9/10 relapses in the corresponding 17 patients occurred within the first 3 years; in the validation cohort, statistical significance was not reached for the same parameter but again, 5/6 relapses were observed within the first 3 years (Fig 3A; S8 Table). Based on this analysis, a first multivariate model was created including hydrophobic mutations and all clinicopathological parameters (patient age and menopausal status, nodal status, tumor size, histology, grade, radiotherapy). High nodal status was the only common independent unfavorable parameter in both cohorts. Resulting hydrophobic mutations remained significant in the training cohort, while a similar trend was observed in the validation cohort when continuous TILs were included in the model (Table 2A and 2B).

**Table 2:** Basic parameter adjustments in the two cohorts.

**A. TILs as a categorical variable were not retained in the model**					
**TRAINING COHORT**	**N patients**	**N events**	**HR**	**95%CI**	**Wald's p**
Menopausal status					
post vs. pre	61 vs. 69	18 vs. 25	0.6	0.32–1.10	0.10
Size					
>2 vs. ≤2 cm	87 vs. 43	32 vs. 11	1.8	0.90–3.58	0.097
Radiotherapy					
yes vs. no	99 vs. 31	31 vs. 12	0.44	0.21–0.90	0.026
Mutated amino acid class					
hydrophobic vs. all other	26 vs. 104	12 vs. 31	2.31	1.17–4.58	0.017
Number of positive nodes					
≥4 vs. 0–3	39 vs. 91	21 vs. 22	3.88	2.01–7.50	<0.001
**VALIDATION COHORT**					
Histo grade					
III vs. I-II	150 vs. 40	32 vs. 15	0.59	0.32–1.09	0.094
Mutated amino acid class					
hydrophobic vs. all other	24 vs. 166	8 vs. 39	1.69	0.78–3.65	0.18
Number of positive nodes					
≥4 vs. 0–3	68 vs. 122	28 vs. 19	2.75	1.52–4.97	<0.001
**B. TILs as a continuous variable were favourable in the validation cohort only**					
**TRAINING COHORT**	**N patients**	**N events**	**HR**	**95%CI**	**Wald's p**
Menopausal status					
post vs. pre	61 vs. 69	18 vs. 25	0.6	0.32–1.10	0.10
Size					
>2 vs. ≤2 cm	87 vs. 43	32 vs. 11	1.8	0.90–3.58	0.097
Radiotherapy					
yes vs. no	99 vs. 31	31 vs. 12	0.44	0.21–0.90	0.026
Mutated amino acid class					
hydrophobic vs. all other	26 vs. 104	12 vs. 31	2.31	1.17–4.58	0.017
Number of positive nodes					
≥4 vs. 0–3	39 vs. 91	21 vs. 22	3.88	2.01–7.50	<0.001
**VALIDATION COHORT**					
Histo grade					
III vs. I-II	126 vs. 37	24 vs. 13	0.57	0.27–1.19	0.13
Mutated amino acid class					
hydrophobic vs. all other	23 vs. 140	8 vs. 29	2.07	0.94–4.59	0.072
Number of positive nodes					
≥4 vs. 0–3	52 vs. 111	19 vs. 18	2.46	1.26–4.79	0.008
TILs continuous (incr. by 5%)	-	-	0.86	0.76–0.98	0.024
Ki67 (incr. by 5%)	-	-	1.06	1.00–1.12	0.053

Based on these results, we partitioned non-LPBC into high and low nodal status; patients with hydrophobic mutations had unfavorable outcome in both cohorts, particularly pronounced in patients with high nodal status (Fig 3B). Distinguishing low and high TILs in non-LPBC (<5% and 5–50% TILs) further supported the unfavorable effect of resulting hydrophobic mutations in the 5–50% TILs subset, in both cohorts (univariate Cox results in S8 Table**)**. This analysis was not possible in patients with <5% TILs, because only two tumors had hydrophobic mutations in this subset in the validation cohort.

**Fig 4:**
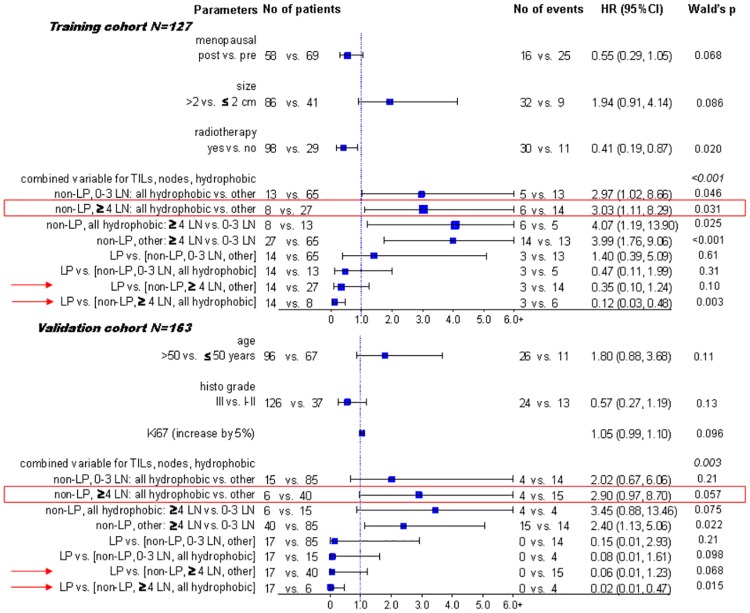
Multivariate analysis results. The unfavorable effect of hydrophobic mutations in patients with high nodal burden (red boxes), especially when compared to LP-TNBC (arrows) was demonstrated in both the training and validation cohorts.

The combined variable including TILs, nodal status and resulting hydrophobic mutations was adjusted for significance with the remaining clinicopathologic variables (Fig 4). The variable retained its significance in both sets, at a first glance because it included nodal status and TILs. Importantly though, all hazard ratios in all subsets were in the same direction, whether significant, marginally significant or non-significant, while the resulting hydrophobic mutations were independently unfavorable prognostic factors in patients with high nodal status, in both the training and validation cohorts. In addition, in both cohorts, LPBC were independently favorable in patients with high nodal burden, especially when compared to patients with the same nodal status and hydrophobic mutations, while only a trend in the same direction was noticed for all other comparisons.

## Discussion

This study confirmed the described complexity and plurality of TNBC mutation profiles [5, 6, 8, 9] in two independent cohorts that were tested with different MPS panels despite that relatively few genes were analyzed. The study also confirmed the favorable prognostic role of increasing TILs density in TNBC in the adjuvant setting [12, 14]. LPBC was highlighted in our previous report [13] as a potentially distinct subgroup of breast tumors with favorable prognosis. Although it has been suggested that LPBC should not be distinguished as a separate category in breast cancer [10], the same effect at the level of relative risk prediction was shown here for the independent training cohort that was not previously analyzed.

Other than described for tumors arising upon exposure to carcinogens or in immunogenic environments [19, 20], and although some TNBC may have mutation load as high as melanomas [33], high TILs density by any approach (continuous or categorical) was not accompanied by higher mutation load or higher numbers of mutated genes, and it was not associated with any structural type of coding mutation the way these are usually studied (missense, indels, nonsense), in neither of the two TNBC cohorts. For the genes analyzed, LP tumors had similar or lower mutation load, as compared to tumors with very low or absent TILs; in the majority of LP tumors no mutations were identified. High TILs in these cases may have been triggered by mutant or chimeric proteins not targeted with the panels; these may also have accumulated as a response to infectious agents the presence of which has been demonstrated in TNBC [34]. Clinically important in the same context is the opposite, that low TILs tumors had mutation load as tumors with higher TILs and LP. This indicates host inefficiency to recognize tumor cells with aberrant proteins and may thus explain the overall worse outcome of such patients.

The novelty here is the investigation of the chemical nature of the resulting mutated amino acids in TNBC genotypes. Although the entire mutation load of the tumors cannot be examined with targeted panels, the presented patterns may be considered representative since the genes mostly affected in TNBC were analyzed. We observed a similar distribution of mutated charged, polar and hydrophobic amino acids in the two cohorts, and similar rates of co-mutation combinations. It will be interesting to define whether these patterns are TNBC-specific or preserved among different tumor types. The small number of mutations in most individual genes did not allow for statistical comparisons. However, mutated amino acid features significantly differed in the most commonly mutated genes, TP53 and PIK3CA. The pattern observed for PIK3CA, i.e., preservation of the charged amino acid class upon mutation may be associated with the low TILs density observed in luminal tumors, where this gene is abundantly mutated [13–15]; the high incidence of changed class and the relatively low rate of preserved charged mutations in TP53 may explain the immunogenicity attributed to mutations of this gene [35, 36].

Among charged, polar and hydrophobic mutated amino acids, only the latter appeared to have an impact on patient outcome. If we accept that hydrophobic epitopes serve in self/non-self discrimination by T cell response [23], and also that increased hydrophobic residues destabilize proteins and turn them prone for proteasome degradation [37], tumors with such mutations would be expected to have dense lymphocytic infiltrations in the normal context. The opposite was observed, in line with tumors being in the escape phase at the time of diagnosis [38, 39]; very low representation of hydrophobic mutations in LP and higher incidence of the same mutations in low TILs tumors. This confirms the inefficiency of host immune response and is compatible with the poor prognosis of such patients. Importantly, we have shown that hydrophobic mutations were associated with poor prognosis in the major subset of early TNBC patients with up to 50% TILs density in their tumors, an effect that was particularly prominent in patients with high nodal status. TNBC patients with high nodal status and non-LP tumors harboring hydrophobic mutations did not benefit from chemotherapy; in fact, this was the only patient subset in both study cohorts with a clear prognostic disadvantage when compared to patients with LP tumors. TILs have been proposed as a marker predictive for response to immune check-point inhibitors [10] and may also be used as triggers for the paused immune system along with conventional therapies [40]. Whether hydrophobic mutations in combination with TILs density may serve as a marker of disabled host immune response is an important clinically relevant question with implications in strategies for the sensitization of the immune system.

A main issue highlighted here is about the TNBC phenotype itself. Discordant ER/PgR and HER2 status has repeatedly been addressed for calling luminal and HER2 positive tumors even between reference laboratories [31], but how often TNBC is miscalled or discordantly called remains unknown. As shown here, IHC-determined basal-like tumors are more consistently triple-negative than non-basal tumors, which has not been previously reported; this is nevertheless compatible with the highest concordance reported between IHC and PAM50 for this phenotype [41]. Phenotype discordance in various depths of the same paraffin block may be the result of technical inconsistency (preanalytical, analytical, IHC scoring and interpretation) but it also may be the result of intra-tumoral heterogeneity [6, 8, 9] and, as shown in preclinical models, of the multipotency of tumor stem cells [42]. In the present series discordance in calling TNBC reflected discordant hormone receptor status. This seems in line with TNBC relating more to the Luminal B subtype at the genomic and gene expression level than to any other breast cancer subtype [43]. In the clinic, TNBC means that the patient will not receive hormonal therapy. If we accept that the TNBC phenotype is heterogeneous or transient within the same tumor, the old practice of treating non-basal TNBC with anti-estrogens should perhaps be reconsidered.

Genomic heterogeneity was revealed as more pronounced for coding mutations than for common polymorphisms. From the technical point, the present comparison of matched samples represents one of the largest series tested with two different panels. As shown, non-coding areas in genes without known alterations in breast cancer such as TERT were fairly preserved in matched samples; by contrast, polymorphisms in genes like MDM2, CDKN2A, EGFR, GATA3 and mutations in frequently affected genes such as in TP53 were frequently not recapitulated. Genomic heterogeneity has been repeatedly demonstrated in TNBC, as reviewed by Ng et al [44] and the present data are further confirmatory in the respect. However, despite the stringent criteria that we applied for accepting variants for inter-sample comparisons with two different MPS panels, technical reasons cannot be excluded for the high degree of the observed genomic heterogeneity in this tumor subset [24].

In conclusion, this study shows that the level of TILs in TNBC is not related to the tumor mutational load and provides evidence suggesting that it may be worthy assessing the chemical nature of mutated amino acids for understanding the functionality of the host immune system according to the type of mutant residues present in the tumor. As in all studies dealing with LP breast cancer, a drawback in the present study is the small number of subsets that emerge for analysis. It appears, however, that LP TNBC is not driven by immunogenic mutations and that tumors with very low TILs may be heavily mutated but not recognized by the host. These findings were confirmed in two independent data sets and patient cohorts and may have implications in the rationale and design of trials involving interventions with immunomodulators. In this context, further validation and elucidation of the evidenced aspects of mutated amino acids seems worthy pursuing.

## Supporting Information

S1 File **Figure A,** REMARK diagram for the study cohorts; **Figure B,** Coding mutations and SNPs in the study cohorts, as assessed with the two panels; **Figure C,** TNBC genotypes in 82 cases with multiple tumor samples; **Figure D,** Probability of DFS for patients in each TNBC cohort according to TILs density.
(DOC)

S1 Table Patients examined in the present study had participated in the listed adjuvant trials by HeCOG. (PDF)

S2 Table Patient and tumor characteristics in the group with paired samples. (PDF)

S3 Table Distribution of mutations in the tumors of the training cohort and of the subsets that emerged according to local/central evaluation in the validation cohort. (PDF)

S4 Table Distribution of mutations in concordant and discordant TNBC for which paired samples were tested with two different panels. (PDF)

S5 Table Mutation characteristics in the training and validation cohorts. (XLS)

S6 Table Associations of TILs and mutated amino acid classes with histological types and with each other in the two study cohorts. (PDF)

S7 Table Comparison of the two study cohorts for the impact of clinicopathological parameters on DFS. (PDF)

S8 Table Univariate Cox analysis for nodal status, TILs density and mutated amino acid class in the two study cohorts. (PDF)
