# Supplementary material for: Tumor Infiltrating Lymphocytes Affect the Outcome of Patients with Operable Triple-Negative Breast Cancer in Combination with Mutated Amino Acid Classes
Source: PLoS One. 2016 Sep 29;11(9):e0163138. doi: 10.1371/journal.pone.0163138 (PMC5042538; doi:10.1371/journal.pone.0163138)
Supplement: S1 File — Figure A, REMARK diagram for the study cohorts; Figure B, Coding mutations and SNPs in the study cohorts, as assessed with the two panels; Figure C, TNBC genotypes in 82 cases with multiple tumor samples; Figure D, Probability of DFS for patients in each TNBC cohort according to TILs density. (DOC) [file pone.0163138.s001.doc]

**Hydrophobic amino acid changes are associated with unfavorable outcome in operable triple-negative breast cancer*.***

*Kotoula V, et al.*

**S1 File : supplementary Figures A - D**

**Figure A: REMARK diagram for the study cohorts.**

**
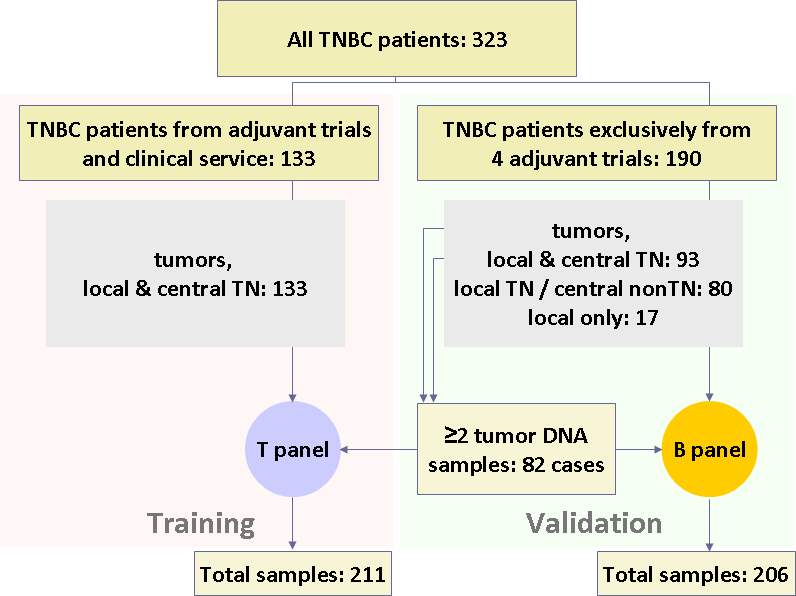
**

TNBC patients with tumors informative upon massively parallel sequencing (MPS) were examined. Local, central: IHC phenotyping in local pathology laboratories (prospective) and upon central testing (retrospective), respectively. Two independent patient groups with tumors collected and processed at two different time points with two different MPS panels served as training and validation cohorts. The total number of samples tested with the T-panel was 211 (133 single tumor samples that were used for associations and outcome analyses in the training cohort and 78 samples paired to tumors in the validation cohort). The total number of samples tested with the B-panel was 206 (190 tumor samples including the 78 tumors with paired samples; 10 additional paired tumor samples within the 82 cases; and, 4 ductal carcinoma in situ). The total number of analyzed samples was 417.

**Figure B: Coding mutations and SNPs in the study cohorts, as assessed with the two panels.**

**a**


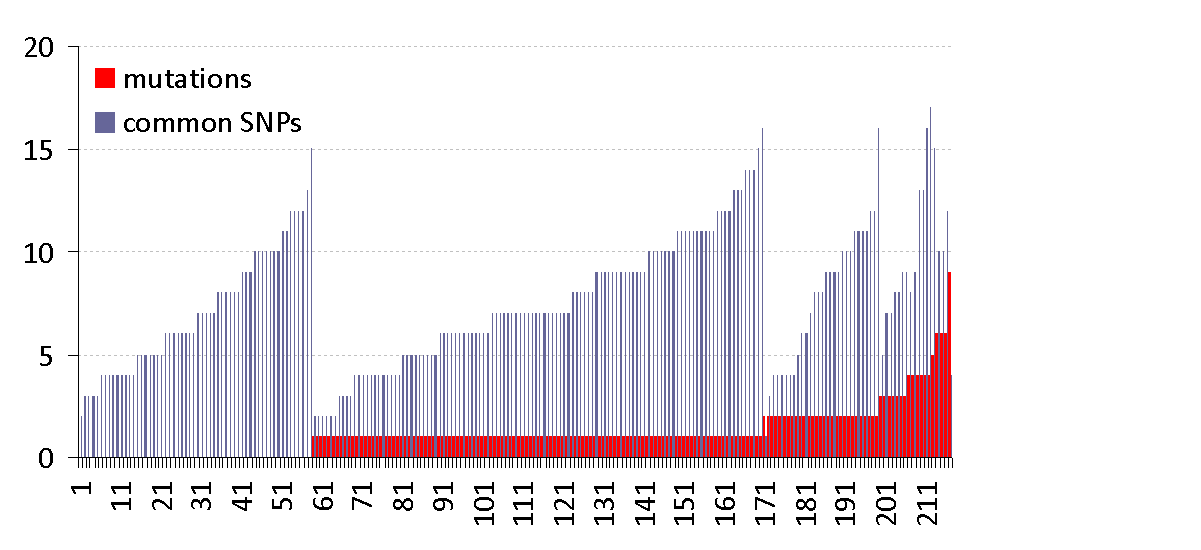


**b**


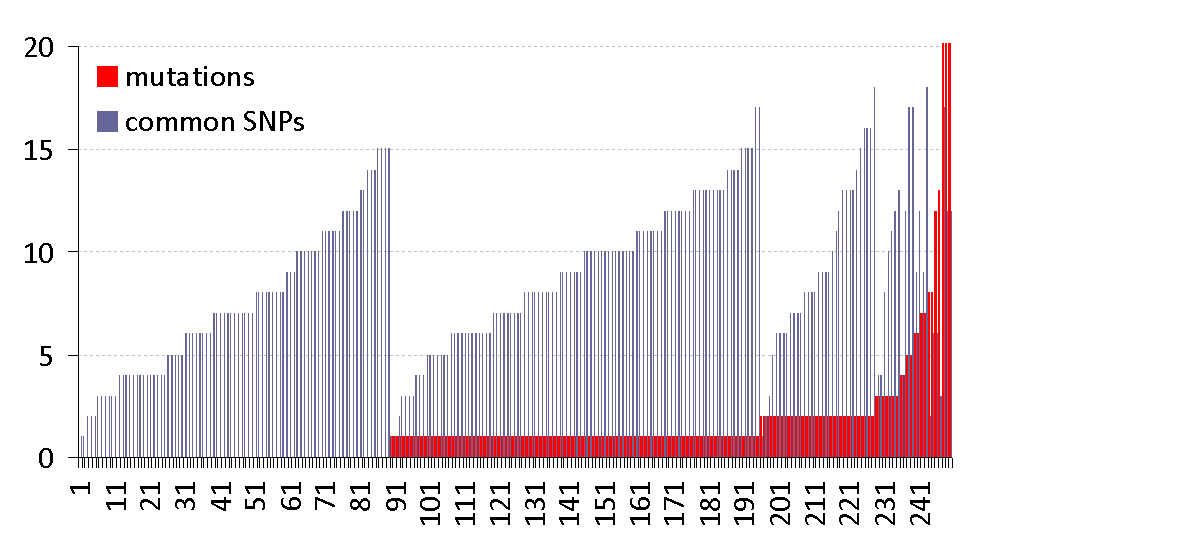


**c**

**d**

1. T-panel results (training cohort and matched samples). Number of mutations per tumor, mean±SD: 1.33±1.17; median: 1; min-max: 1-9.
2. B-panel results (validation cohort). Number of mutations per tumor, mean±SD: 1.48±5.6; median: 1; min-max: 1-60. The Y-axis is truncated at 20, for pattern comparison with the T-panel. In 2 cases, the number of mutations was >20 (arrows).
3. Distribution of mutated genes in the 133 cases of the training cohort.
4. Distribution of mutated genes in the 190 cases of the validation cohort. There were 2 extremely hypermutated tumors (black dots) due to which the list of mutated genes appears longer in this cohort as compared to the training cohort in C.

In both cohorts, LPBC, i.e., tumors with >50% stromal tumor infiltrating lymphocytes (TILs) had mostly TP53 mutations but their mutation profiles did not differ as compared to non-LPBC.

**Figure C. TNBC genotypes in 82 cases with multiple tumor samples.**

**
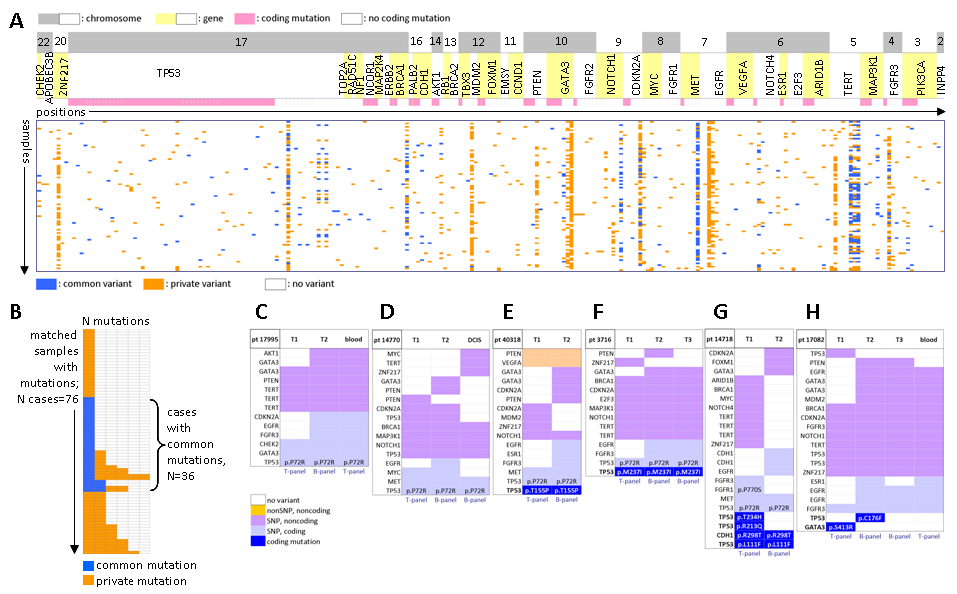
**

Variants at genomic positions covered for all samples by >100 times were analyzed.

**A.** Comparison of all variants in matched tumor samples. The majority were common SNPs, some of which, e.g., in GATA3, EGFR, MDM2, and ZNF217 were extremely poorly preserved between samples.

**B.** Few cases with at least one common mutation were observed.

**C-H:** Example genotypes for cases with matched samples. T1, T2, T3 correspond to different tumor samples from the same or from different tumor blocks. Peripheral blood and carcinoma in situ (DCIS) samples were also compared for eligible positions. The panel applied for each sample is indicated at the bottom of each lane. Intra-tumoral genotype diversity concerned both SNPs and coding mutations.

Out of 1372 variants adequately covered with both panels, 370 were common in matched samples; the rest were present in individual samples and were characterized as private. SNPs, especially those in the TERT gene, were most frequently conserved (**A**). Mutations were present in 76/82 cases (92.8%). Among these, coding mutations were common in 36/76 cases (50%). In 34/36 cases with concordant mutations, these were found in TP53. The rest of the samples demonstrated private mutations (**B**), while no two tumors had identical genotypes in matched samples (**C-H**). The incidence of mutated TP53 and PIK3CA did not differ in cases with discordant and concordant central TNBC phenotypes (**Table S4**).

**Figure D: Probability of DFS for patients in each TNBC cohort according to TILs density.**


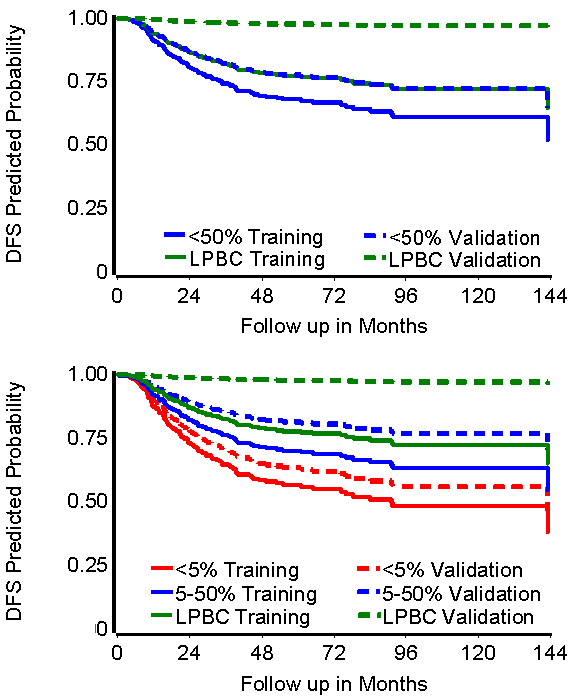


The risk for relapse was overall higher in the training cohort. Patients in the worst performing category in the validation cohort had the same risk for relapse as patients in the best performing category in the training cohort. However, the same trend for favorable LP tumors was present in both cohorts.
