# Supplementary material for: Tumor Infiltrating Lymphocytes Affect the Outcome of Patients with Operable Triple-Negative Breast Cancer in Combination with Mutated Amino Acid Classes
Source: PLoS One. 2016 Sep 29;11(9):e0163138. doi: 10.1371/journal.pone.0163138 (PMC5042538; doi:10.1371/journal.pone.0163138)
Supplement: S2 Table — (PDF) [file pone.0163138.s003.pdf]

TABLE S2: Patient and tumor characteristics in the group with paired samples

	TNBC both
**Patients**	
N	82
**Age (years)**	
Mean (SD)	53.4 (11.6)
Median	55,4
Min-Max	21-74
**Ki67**	
Mean (SD)	55.9 (31.3)
Median	57,5
Min-Max	0-100
	**N (%)**
**Age (years)**	
≤50	30 (36.6)
>50	52 (63.4)
**Menopausal status**	
Postmenopausal	48 (58.6)
Premenopausal	34 (41.4)
**Tumor size**	
≤2	29 (35.4)
>2	53 (64.6)
**Positive lymph nodes**	
0-3	58 (70.8)
≥4	24 (29.2)
**Histological grade**	
I	3 (3.6)
II	8 (9.8)
III	71 (86.6)
**Histological type**	
Medullary	9 (11.0)
NST	54 (65.8)
Other	19 (23.2)
**Surgery (binary)**	
MRM	39 (47.6)
Other	43 (52.4)
**Hormonotherapy**	
No	71 (86.6)
Yes	11 (13.4)
**Radiotherapy**	
No	21 (25.6)
Yes	61 (74.4)
**ER/PgR/HER2 local**	
either positive	4 (4.8)
TNBC	78 (95.2)
**ER/PgR/HER2 central**	
either positive	23 (28.1)
TNBC	54 (65.8)
At least one not assessed	5 (6.1)
**Basal**	
Yes	67 (83.8)
No	13 (16.2)
**Survival data**	
Median FU in months	70
N of valid cases	82
Deaths, N	16
Event free at 3 years, %	91,3
Event free at 5 years, %	80,4
Relapse, N	22
Event free at 3 years, %	81,4
Event free at 5 years, %	71,1

Notes: N: number; MRM: modified radical mastectomy;
NST: non-specific type; FU: follow-up
