# Supplementary material for: Tumor Infiltrating Lymphocytes Affect the Outcome of Patients with Operable Triple-Negative Breast Cancer in Combination with Mutated Amino Acid Classes
Source: PLoS One. 2016 Sep 29;11(9):e0163138. doi: 10.1371/journal.pone.0163138 (PMC5042538; doi:10.1371/journal.pone.0163138)
Supplement: S3 Table — (PDF) [file pone.0163138.s004.pdf]

TABLE S3: Distribution of mutations in the tumours of the training cohort and of the subsets that emerged according to local/central evaluation in the validation cohort.

	Training, concordant TNBC, 94/133 (70.7%) with mutations		Validation, concordant TNBC, 61/93 (74.2%) with mutations		Validation*, local TN / central nonTN, 56/80 (70.0%) with mutations		
	**N mutated tumors**	***% among mutated tumors***	**N mutated tumors**	***% among mutated tumors***	**N mutated tumors**	***% among mutated tumors***	**Chi sq p-value^**
**AKT1**	2	*2,1*	4	*6,6*	3	*5,4*	
**APOBEC3B**	0		2	*3,3*	1	*1,8*	
**ARID1B**	2	*2,1*	3	*4,9*	1	*1,8*	
**BRCA1**	2	*2,1*	1	*1,6*	3	*5,4*	
**BRCA2**	0		0		1	*1,8*	
**CCND1**	5	*5,3*	0		2	*3,6*	
**CDH1**	12	*12,8*	3	*4,9*	3	*5,4*	
**CDKN2A**	0		1	*1,6*	1	*1,8*	
**CHEK2**	0		0		1	*1,8*	
**E2F3**	0		1	*1,6*	1	*1,8*	
**EGFR**	2	*2,1*	2	*3,3*	3	*5,4*	
**ESR1**	3	*3,2*	0		0		
**FGFR1**	0		0		1	*1,8*	
**FGFR2**	0		0		3	*5,4*	
**FGFR3**	0		0		1	*1,8*	
**FOXM1**	0		2	*3,3*	2	*3,6*	
**GATA3**	2	*2,1*	5	*8,2*	2	*3,6*	
**MAP2K4**	2	*2,1*	2	*3,3*	4	*7,1*	
**MAP3K1**	1	*1,1*	3	*4,9*	3	*5,4*	
**MDM2**	0		2	*3,3*	1	*1,8*	
**MET**	0		3	*4,9*	0		
**MYC**	0		0		0		
**NCOR1**	0		2	*3,3*	2	*3,6*	
**NF1**	0		0		1	*1,8*	
**NOTCH1**	0		0		1	*1,8*	
**NOTCH4**	1	*1,1*	0		1	*1,8*	
**PALB2**	1	*1,1*	2	*3,3*	1	*1,8*	
**PIK3CA**	11	*11,7*	14	*23,0*	16	*28,5*	<0.001
**PTEN**	3	*3,2*	4	*6,6*	4	*7,1*	
**RAD51C**	0		1	*1,6*	1	*1,8*	
**RB1**	0		1	*1,6*	2	*3,6*	
**TBX3**	1	*1,1*	2	*3,3*	4	*7,1*	
**TP53**	77	*81,9*	43	*70,5*	34	*60,7*	<0.001
**VEGFA**	1	*1,1*	0		1	*1,8*	
**ZNF703**	0		1	*1,6*	1	*1,8*	

*: mutation rates for the 17 cases that could not be re-assessed centrally are not shown; ^: comparison between tratining and local TN / central nonTN
