# Supplementary material for: Tumor Infiltrating Lymphocytes Affect the Outcome of Patients with Operable Triple-Negative Breast Cancer in Combination with Mutated Amino Acid Classes
Source: PLoS One. 2016 Sep 29;11(9):e0163138. doi: 10.1371/journal.pone.0163138 (PMC5042538; doi:10.1371/journal.pone.0163138)
Supplement: S4 Table — (PDF) [file pone.0163138.s005.pdf]

TABLE S4: Distribution of mutations in concordant and discordant TNBC for which paired samples were tested with two different panels. These 82 tumors were tested twice centrally, at different time points; in 23 out of these, discordant phenotypes were obtained (TN / nonTN). TP53 and PIK3CA mutation rates were remarkably consistent in the two groups.

**gene**	**heterochronous concordant TNBC phenotype**	**heterochronous discordant TNBC phenotype**
AKT1	2	none
ARID1B	3	none
BRCA1	1	1
CDH1	4	2
CDKN2A	1	1
ESR1	none	1
FGFR2	none	1
GATA3	3	2
MAP2K4	none	1
MAP3K1	5	none
MET	1	none
NCOR1	4	none
PALB2	none	1
PIK3CA	14 (12.4%)	5 (10.9%)
PTEN	2	2
TP53	72 (63.7%)	32 (62.9%)
VEGFA	1	1
all mutations	113	46
