# Supplementary material for: Tumor Infiltrating Lymphocytes Affect the Outcome of Patients with Operable Triple-Negative Breast Cancer in Combination with Mutated Amino Acid Classes
Source: PLoS One. 2016 Sep 29;11(9):e0163138. doi: 10.1371/journal.pone.0163138 (PMC5042538; doi:10.1371/journal.pone.0163138)
Supplement: S6 Table — (PDF) [file pone.0163138.s007.pdf]

TABLE S6: Associations of TILs and mutated amino acid classes with histological types and with each other in the two study cohorts

TRAINING COHORT

Histo_Classification By TILs 3 scale	<5	5 to 50	>50	total	Chi-sq p-value
apocrine	0	1	0	1	0.79
invasive lobular	1	2	0	3	
medullary	0	3	2	5	
medullary-like	0	1	0	1	
metaplastic	1	4	0	5	
invasive ductal, NST	16	81	12	109	
Other	1	5	0	6	
total	19	97	14	130	

Histological type By charged	NO	YES	total	
apocrine	1	0	1	0.42
invasive lobular	3	0	3	
medullary	5	0	5	
medullary-like	1	0	1	
metaplastic	3	2	5	
invasive ductal, NST	85	27	112	
Other	3	3	6	
total	101	32	133	

Histological type By polar	NO	YES	total	
apocrine	1	0	1	0.33
invasive lobular	2	1	3	
medullary	2	3	5	
medullary-like	0	1	1	
metaplastic	5	0	5	
invasive ductal, NST	72	40	112	
Other	3	3	6	
total	85	48	133	

Histological type By hydrophobic	NO	YES	total	
apocrine	1	0	1	0.86
invasive lobular	2	1	3	
medullary	3	2	5	
medullary-like	1	0	1	
metaplastic	4	1	5	
invasive ductal, NST	90	22	112	
Other	4	2	6	
total	105	28	133	

charged By TILs 3scale	<5	5 to 50	>50	total	
NO	15	70	13	98	0.23
YES	4	27	1	32	
total	19	97	14	130	

polar By TILs 3scale	<5	5 to 50	>50	total	
NO	13	61	8	82	0.80
YES	6	36	6	48	
total	19	97	14	130	

hydrophobic By TILs 3scale	<5	5 to 50	>50	total	
NO	10	84	10	104	**0.002**
YES	9	13	4	26	
total	19	97	14	130	

MUT amino acid class By TILs 3 scale	<5	5 to 50	>50	total	
all	1	0	1	2	**0.039**
charged	0	15	0	15	
charged&polar	2	7	0	9	
hydro&charged	1	3	0	4	
hydro&polar	1	3	1	5	
hydrophobic	6	7	2	15	
no mutation	4	29	6	39	
nonsense	2	5	0	7	
polar	2	28	4	34	
total	19	97	14	130	

VALIDATION COHORT

Histological type By TILs 3 scale	<5	5 to 50	>50	total	Chi-sq p-value
comedo	0	1	0	1	**<0.001**
invasive ductal. NST	26	101	10	137	
invasive lobular	5	3	1	9	
medullary	0	8	8	16	
mixed	2	3	0	5	
Other	3	8	0	11	
total	36	124	19	179	

Histological type By charged	NO	YES	total	
comedo	0	1	1	**0.014**
invasive ductal. NST	118	30	148	
invasive lobular	6	3	9	
medullary	15	1	16	
mixed	2	3	5	
Other	6	5	11	
total	147	43	190	

Histological type By polar	NO	YES	total	
comedo	1	0	1	0.35
invasive ductal. NST	102	46	148	
invasive lobular	6	3	9	
medullary	14	2	16	
mixed	5	0	5	
Other	9	2	11	
total	137	53	190	

Histological type By hydrophobic	NO	YES	total	
comedo	1	0	1	0.95
invasive ductal. NST	123	25	148	
invasive lobular	7	2	9	
medullary	14	2	16	
mixed	4	1	5	
Other	10	1	11	
total	159	31	190	

charged By TILs 3scale	<5	5 to 50	>50	total	
NO	26	96	17	139	0.34
YES	10	28	2	40	
total	36	124	19	179	

polar By TILs 3scale	<5	5 to 50	>50	total	
NO	29	84	16	129	0.15
YES	7	40	3	50	
total	36	124	19	179	

hydrophobic By TILs 3scale	<5	5 to 50	>50		
NO	31	100	17	148	0.53
YES	5	24	2	31	
total	36	124	19	179	

MUT amino acid class By TILs 3scale	<5	5 to 50	>50	total	
all	0	7	0	7	0.36
charged	7	13	1	21	
charged&polar	2	6	1	9	
hydro & charged	1	2	0	3	
hydro & polar	2	1	1	4	
hydrophobic	2	14	1	17	
no mutation	14	36	10	60	
nonsense	5	19	4	28	
polar	3	26	1	30	
total	36	124	19	179	

BOTH COHORTS

Histological type By TILs 3scale	<5	5 to 50	>50	total	Chi-sq p-value
apocrine	0	1	0	1	**0.002**
comedo	0	1	0	1	
invasive lobular	6	5	1	12	
medullary	0	11	10	21	
medullary-like	0	1	0	1	
metaplastic	1	4	0	5	
invasive ductal. NST	42	182	22	246	
mixed	2	3	0	5	
Other	4	13	0	17	
total	55	221	33	309	

Histological type By charged	NO	YES	total	
apocrine	1	0	1	**0.004**
comedo	0	1	1	
invasive lobular	9	3	12	
medullary	20	1	21	
medullary-like	1	0	1	
metaplastic	3	2	5	
invasive ductal. NST	203	57	260	
mixed	2	3	5	
Other	9	8	17	
total	248	75	323	

Histological type By polar	NO	YES	total	
apocrine	1	0	1	0.32
comedo	1	0	1	
invasive lobular	8	4	12	
medullary	16	5	21	
medullary-like	0	1	1	
metaplastic	5	0	5	
invasive ductal. NST	174	86	260	
mixed	5	0	5	
Other	12	5	17	
total	222	101	323	

Histological type By hydrophobic	NO	YES	total	
apocrine	1	0	1	>0.99
comedo	1	0	1	
invasive lobular	9	3	12	
medullary	17	4	21	
medullary-like	1	0	1	
metaplastic	4	1	5	
invasive ductal. NST	213	47	260	
mixed	4	1	5	
Other	14	3	17	
total	264	59	323	

charged By TILs 3scale	<5	5 to 50	>50	total	
NO	41	166	30	237	0.12
YES	14	55	3	72	
total	55	221	33	309	

polar By TILs 3scale	<5	5 to 50	>50	total	
NO	42	145	24	211	0.28
YES	13	76	9	98	
total	55	221	33	309	

hydrophobic By TILs 3scale	<5	5 to 50	>50	total	
NO	41	184	27	252	0.32
YES	14	37	6	57	
total	55	221	33	309	

MUT amino acid class By TILs 3scale	<5	5 to 50	>50	total	
all	1	7	1	9	0.34
charged	7	28	1	36	
charged&polar	4	13	1	18	
hydro & charged	2	5	0	7	
hydro & polar	3	4	2	9	
hydrophobic	8	21	3	32	
no mutation	18	65	16	99	
nonsense	7	24	4	35	
polar	5	54	5	64	
total	55	221	33	309	
