# Supplementary material for: Tumor Infiltrating Lymphocytes Affect the Outcome of Patients with Operable Triple-Negative Breast Cancer in Combination with Mutated Amino Acid Classes
Source: PLoS One. 2016 Sep 29;11(9):e0163138. doi: 10.1371/journal.pone.0163138 (PMC5042538; doi:10.1371/journal.pone.0163138)
Supplement: S7 Table — (PDF) [file pone.0163138.s008.pdf]

TABLE S7: Comparison of the two study cohorts for the impact of clinicopathological parameters on DFS

		TRAINING cohort, n patients: 133					VALIDATION cohort, n patients: 190				
Parameter	Categories	N patients	N events	HR	95% CI	Wald's p	N patients	N events	HR	95% CI	Wald's p
Age											
	>50 vs. ≤50	65 vs. 68	20 vs. 24	0.83	0.46-1.51	0.55	116 vs. 74	33 vs. 14	1.61	0.86-3.01	0.14
Histological grade											
	III vs. I-II	111 vs. 22	39 vs. 5	1.74	0.68-4.41	0.24	150 vs. 40	32 vs. 15	0.54	0.29-1.00	0.051
Histological type											
	all other vs. NST	21 vs. 112	5 vs. 39	0.63	0.25-1.59	0.32	42 vs. 148	9 vs. 38	0.82	0.39-1.69	0.59
Hormonotherapy											
	Yes vs. No	26 vs. 107	13 vs. 31	1.7	0.89-3.25	0.11	28 vs. 160	6 vs. 40	0.64	0.27-1.54	0.32
Menopausal status											
	post vs. pre	64 vs. 69	19 vs. 25	0.78	0.43-1.42	0.43	103 vs. 87	27 vs. 20	1.13	0.63-2.01	0.68
Radiotherapy											
	Yes vs. No	99 vs. 34	31 vs. 13	0.76	0.40-1.46	0.42	142 vs. 44	35 vs. 11	1.01	0.51-2.00	0.97
Tumor size											
	>2 vs. ≤2	89 vs. 43	33 vs. 11	1.81	0.92-3.59	0.088	118 vs. 72	36 vs. 11	2.19	1.12-4.31	**0.023**
Nodes ≥4 vs. 0-3		39 vs. 91	21 vs. 22	2.81	1.54-5.11	**0.001**	68 vs. 122	28 vs. 19	2.78	1.55-5.00	**0.001**
TILs cut off at 50%, LPBC vs. non-LPBC		14 vs. 116	3 vs. 39	0.58	0.18-1.87	0.36	19 vs. 160	0 vs. 41	0.09	0.01-1.52	0.095
TILs, 3 scale											
LPBC vs. TILs 5-50%		14 vs. 97	3 vs. 31	0.62	0.19-2.03	0.43	19 vs. 124	0 vs. 26	0.11	<0.01-1.93	0.13
LPBC vs. TILs <5%		14 vs. 22	3 vs. 10	0.39	0.11-1.42	0.15	19 vs. 47	0 vs. 21	0.05	<0.01-0.84	**0.037**
TILs 5-50% vs. TILs <5%		97 vs. 22	31 vs. 10	0.63	0.31-1.29	0.2	124 vs. 47	26 vs. 21	0.43	0.24-0.77	**0.004**
TILs continuous (incr. by 5%)		-	-	0.92	0.84-1.01	0.085	-	-	0.86	0.76-0.97	**0.011**

HR: hazard ratio; bold letters: statistically significant results
