# Supplementary material for: Tumor Infiltrating Lymphocytes Affect the Outcome of Patients with Operable Triple-Negative Breast Cancer in Combination with Mutated Amino Acid Classes
Source: PLoS One. 2016 Sep 29;11(9):e0163138. doi: 10.1371/journal.pone.0163138 (PMC5042538; doi:10.1371/journal.pone.0163138)
Supplement: S8 Table — (PDF) [file pone.0163138.s009.pdf]

TABLE S8: Univariate Cox analysis for nodal status, TILs density and mutated amino acid class in the two study cohorts

	TRAINING COHORT					VALIDATION COHORT				
	N patients	N events	HR	95% CI	Wald's p	N patients	N events	HR	95% CI	Wald's p
hydrophobic vs. no mutation	17 vs. 39	10 vs. 7	5.13	1.95-13.52	**<0.001**	17 vs. 63	6 vs. 13	1.81	0.69-4.78	0.23
hydrophobic vs. all other mutation	17 vs. 77	10 vs. 27	2.43	1.17-5.03	**0.017**	17 vs. 110	6 vs. 28	1.49	0.61-3.59	0.38
all other mut vs. no mutation	77 vs. 39	27 vs. 7	2.12	0.92-4.86	0.078	110 vs. 63	28 vs. 13	1.22	0.63-2.36	0.55
non-LPBC, 0-3 LN (all hydrophobic vs. all other)	13 vs. 65	5 vs. 13	2.72	0.97-7.67	0.058	16 vs. 91	4 vs. 14	1.63	0.54-4.97	0.39
non-LPBC, ≥4 LN (all hydrophobic vs. all other)	8 vs. 27	6 vs. 14	2.22	0.85-5.82	0.11	6 vs. 47	4 vs. 19	2.1	0.71-6.21	0.18
TILs 5-50% (hydrophobic vs. all other)	7 vs. 90	4 vs. 27	2.76	0.96-7.92	**0.059**	14 vs. 110	6 vs. 20	2.69	1.08-6.71	**0.034**
TILs 0-5% (hydrophobic vs. all other)	6 vs.13	5 vs. 3	11.99	2.76-52.16	**<0.001**	2 vs. 34		not comparable		
continuous TILs (5% increments), hydrophobic	-	-	0.84	0.65-1.07	0.16	-	-	0.94	0.71-1.23	0.64
continuous TILs, all other	-	-	0.95	0.86-1.05	0.29	-	-	0.84	0.73-0.96	**0.012**
conitnuous TILs, 0-3 LN, all hydrophobic	-	-	0.84	0.62-1.14	0.25	-	-	0.9	0.62-1.29	0.56
conitnuous TILs, 0-3 LN, all other	-	-	1	0.89-1.13	0.97	-	-	0.87	0.72-1.06	0.18
conitnuous TILs, ≥4 LN, all hydrophobic	-	-	0.81	0.58-1.12	0.2	-	-	0.92	0.71-1.19	0.51
conitnuous TILs, ≥4 LN, all other	-	-	0.9	0.75-1.08	0.27	-	-	0.83	0.69-1	0.053
CDH1 mut vs. non-mut	12 vs. 121	5 vs. 39	1.22	0.48-3.09	0.68	7 vs. 183	3 vs. 44	1.5	0.46-4.86	0.5
GATA3 mut vs. non-mut	2 vs. 131		not comparable			7 vs. 183	1 vs. 46	0.46	0.06-3.34	0.44
PIK3CA mut vs. non-mut	11 vs. 122	5 vs. 39	1.61	0.63-4.08	0.32	33 vs. 157	10 vs. 37	1.32	0.65-2.66	0.44
PTEN mut vs. non-mut	3 vs. 130		not comparable			8 vs. 182	3 vs. 44	2.03	0.63-6.56	0.24
TP53 mut vs. non-mut	77 vs. 56	27 vs. 17	1.2	0.65-2.21	0.55	86 vs. 104	27 vs. 20	1.73	0.97-3.08	0.065
